# Pronerve Growth Factor Induces Angiogenesis via Activation of TrkA: Possible Role in Proliferative Diabetic Retinopathy

**DOI:** 10.1155/2013/432659

**Published:** 2013-08-12

**Authors:** Sally L. Elshaer, Mohammed A. Abdelsaid, Ahmad Al-Azayzih, Parag Kumar, Suraporn Matragoon, Julian J. Nussbaum, Azza B. El-Remessy

**Affiliations:** ^1^Center for Pharmacy and Experimental Therapeutics, University of Georgia, 1120 15th Street HM-1200, Augusta, GA 30912, USA; ^2^Culver Vision Discovery Institute, Georgia Reagents University, Augusta, GA 30912, USA; ^3^Charlie Norwood VA Medical Center, Augusta, GA 30912, USA; ^4^Department of Physiology, Georgia Reagents University, Augusta, Georgia 30912, USA; ^5^Pharmacy Department, National Institutes of Health Clinical Center, Bethesda, MD 20892, USA

## Abstract

Proliferative diabetic retinopathy (PDR) is the leading cause of blindness in working age Americans. We demonstrated that diabetes disturbs the homeostasis of nerve growth factor (NGF) resulting in accumulation of its precursor proNGF. Increases in proNGF were positively correlated with progression of diabetic retinopathy, having the highest level in ocular fluids from PDR patients compared to nondiabetic patients. Here, we attempted to evaluate the contribution and the possible mechanism of proNGF to PDR. The angiogenic response of aqueous humor samples from PDR patients was examined in human retinal endothelial cells in the presence or absence of anti-proNGF antibody. Additional cultures were treated with mutant-proNGF in the presence of specific pharmacological inhibitors of TrkA and p75^NTR^ receptors. PDR-aqueous humor samples exerted significant angiogenic response including cell proliferation, migration, and alignment into tube-like structures. These effects were significantly reduced by anti-proNGF antibody but not by IgG. Treatment of retinal endothelial cells with mutant-proNGF activated phosphorylation of TrkA and p38MAPK; however, it did not alter p75^NTR^ expression. Inhibition of TrkA but not p75^NTR^ significantly reduced mutant-proNGF-induced cell proliferation, cell migration, and tube formation. Taken together, these results provide evidence that proNGF can contribute to PDR at least in part via activation of TrkA.

## 1. Introduction

Diabetic retinopathy (DR) is the leading cause of blindness among working aged adults in the US. It affects 80% of individuals with a 10-year history of diabetes, adding 63,000 new cases of DR each year [1]. DR is characterized by neuro- and vascular degeneration that eventually lead to ischemia and subsequent release of angiogenic growth factors including vascular endothelial growth factor (VEGF) into the vitreous cavity resulting in retinal neovascularization and proliferative diabetic retinopathy (PDR) [2, 3]. PDR is characterized by vitreous hemorrhage, neovascular glaucoma, and tractional retinal detachment, which can result in visual loss [4]. Current treatment options for PDR include laser photocoagulation and anti-VEGF ocular injection, which are invasive and limited by side effects. Repeated injections of anti-VEGF can deprive the retina from the survival actions of VEGF on neurons and vasculature (reviewed in [2, 5]). Therefore, there is a great need to identify contributing factors in PDR other than VEGF; in the hope of devising treatments that will preserve both retina vasculature and neuronal function.

Diabetes-induced oxidative stress disturbs retinal homeostasis by activating glial cells, reducing neurotrophic support, and increasing proinflammatory cytokines including VEGF, IL-1*β*, and TNF-*α* [6, 7]. In addition to these known growth factors, recent findings using ocular fluids from diabetic patients and experimental models of diabetes suggest that neurotrophins including nerve growth factor (NGF) are emerging as critical mediators of DR [5, 8–11]. NGF is produced by neurons and many nonneuronal cell types such as immune cells, inflammatory cells, and smooth muscle cells [12]. It was originally characterized by its ability to stimulate growth, differentiation, and survival of neurons; however, NGF appears as a pleiotropic modulator of wound healing and reparative angiogenesis [13–15]. NGF activates two different receptors including the high affinity tropomyosin-related receptor A (TrkA), which is a tyrosine kinase, and the low affinity p75^NTR^ neurotrophin receptors (p75^NTR^) [16]. Previous studies demonstrated that the angiogenic response of NGF was mediated via activation of TrkA [15, 17, 18]. 

NGF is synthesized and secreted by glial cells as the precursor proNGF which is cleaved, by furin intracellularly and by the matrix metalloproteinase-7 (MMP-7) extracellularly, to generate mature NGF [19]. Our studies showed that diabetes-induced peroxynitrite formation impairs maturation of NGF, leading to accumulation of its precursor proNGF both in experimental models and in clinical diabetes [10, 11]. In these studies, we used specific antibodies to detect NGF (13 kDa) and proNGF (32 kDa) rather than ELISA assays that detect both NGF and proNGF. Our results showed that increases in proNGF positively correlated with progression of the disease where ocular fluids from PDR patients showed the higher level of proNGF (5-fold) and lower level of NGF (65% less) compared to nondiabetic samples [10]. Interestingly, earlier studies utilizing ELISA showed higher NGF levels in PDR patients than in controls and nonproliferative diabetic retinopathy (NPDR) patients [9]. Because many NGF antibodies can detect both NGF and proNGF, these increases may reflect the combined presence of both NGF and proNGF. Based on these observations, it appears that proNGF may contribute to development and progression of proliferative diabetic retinopathy clinically. Here, we attempted to evaluate the specific contribution of proNGF to angiogenic response of ocular fluids from PDR patients within retinal endothelial cells and to elucidate the possible role of TrkA and p75^NTR^ in mediating the angiogenic signal. 

## 2. Materials and Methods

### 2.1. Human Aqueous Humor Samples

Human specimens were obtained with the Institutional Review Board approval from the Human Assurance Committee at Georgia Regents University. Aqueous humor samples were collected from Eye Clinic at Georgia Regents University from patients undergoing intravitreal injections and were identified as being from patients with PDR.  Table 1 shows the clinical characteristics of participants providing aqueous humor samples.

### 2.2. Cell Culture

Human retinal endothelial (HRE) cells and cell culture medium were purchased from Cell Systems Corporations (Kirkland, WA, USA) and VEC Technologies (Rensselaer, NY, USA), respectively. Experiments were performed using cells between passages (4–6) at 37°C in a humidified atmosphere of 5% CO_2_. Cells were switched to serum free medium containing 50% of MCDB131 complete medium (VEC Technology, Rensselaer NY) overnight prior to stimulation with aqueous humor samples (10 *μ*L/mL) from various patients in the presence or absence of either anti-proNGF antibody or isotope control rabbit IgG (1 *μ*g/mL). Mutant (cleavage-resistant) proNGF protein and anti-proNGF antibody were purchased from Alomone Labs (Jerusalem, Israel) and IgG was purchased from Cell Signaling Technology (Danvers, MA). For proNGF studies, bovine retinal endothelial (BRE) cells were cultured as described previously [20]. Cells from passages 4 to 8 were used in all experiments. Cells were maintained in M199 supplemented with 10% fetal bovine serum, 10% CS-C complete medium, 2 mM glutamine, 100 U/mL penicillin, and 100 *μ*g/mL streptomycin at 37°C in a humidified CO_2_ incubator. Cells were stimulated with proNGF (50 ng/mL) in the presence or absence of either TrkA antagonist, K252a (0.1 *μ*M) from Calbiochem/EMD Bioscience (La Jolla, CA) or p75^NTR^ selective p75 antagonist A (C30–35, 20 *μ*M), a kind of gift from Uri Saragovi, McGill University, Canada [21].

### 2.3. Endothelial Cell Migration

HRECs and BRECs were grown to confluence and then were wounded with a single sterile cell scraper of constant diameter as described previously [22]. Images of wounded areas were taken immediately after adding the treatment and after 18 h and % cell migration was calculated. Each condition was verified in triplicate and was repeated using independent cultures.

### 2.4. Tube Formation

Tube formation assay was performed using growth factor-reduced Matrigel (BD Biosciences) as described previously [23, 24]. HRE cells and BRE cells were counted and plated at 2 × 10^4^ cells/mL with Matrigel in a 96-well plate. Eighteen hours later, images of the tube-like structures were captured and analyzed using Zeiss Axiovert microscope software. Each condition was verified in triplicate and was repeated using independent cultures.

### 2.5. Endothelial Cell Proliferation

Cells were seeded at a density of 0.5 × 10^5^/mL, switched to medium containing 0.5% FBS, and incubated overnight. Cells were incubated with and without various treatments in medium containing 0.2% FBS for 24 h. After trypsinization, the cell number was determined using a hemocytometer [23, 24]. Each condition was verified in triplicate and was repeated using independent cultures.

### 2.6. Western Blot Analysis

For analysis of protein, bovine retinal endothelial cells were homogenized in a modified RIPA buffer from (Millipore, Billerica MA), in the presence of Protease inhibitor cocktail (Sigma Aldrich, St. Louise MO), and Halt Phosphatase inhibitor (Thermo Scientific, Rockford IL). Total protein concentrations were measured using Bio-Rad protein assay. Protein samples (20 *μ*g) were separated by 8% sodium dodecyl sulfate-polyacrylamide gel electrophoresis, transferred to nitrocellulose membrane, and probed with the following antibodies: TrkA (Calbiochem/EMD Bioscience (La Jolla, CA)), phospho-TrkA (Santa Cruz Biotechnology, Santa Cruz Biotechnology, Dallas TX), p38MAPK and phospho-p38 MAPK (Cell Signaling Technology, Danvers, MA, USA), rabbit anti-p75^NTR^ provided by Dr. Bruce Carter Vanderbilt University School of Medicine, Nashville, TN, USA) and tubulin (abcam, Cambridge, MA, USA) followed by secondary horseradish peroxidase-conjugated sheep anti-rabbit antibody and enhanced chemiluminescence (Pierce/Thermo Scientific, Rockford IL). The films were subsequently scanned and band intensity was quantified using densitometry software (fluorchem FC2) and expressed as relative optical density to controls.

### 2.7. Data Analysis

The results are expressed as mean ± SEM. Differences between experimental groups were evaluated by ANOVA and the significance of differences between groups was assessed by the post hoc test (Fisher's protected least significant difference) when indicated. Significance was defined as *P* < 0.05.

## 3. Results 

### 3.1. PDR-Aqueous Humor Stimulates Cell Migration in a ProNGF-Dependent Manner

**Figure 1:**
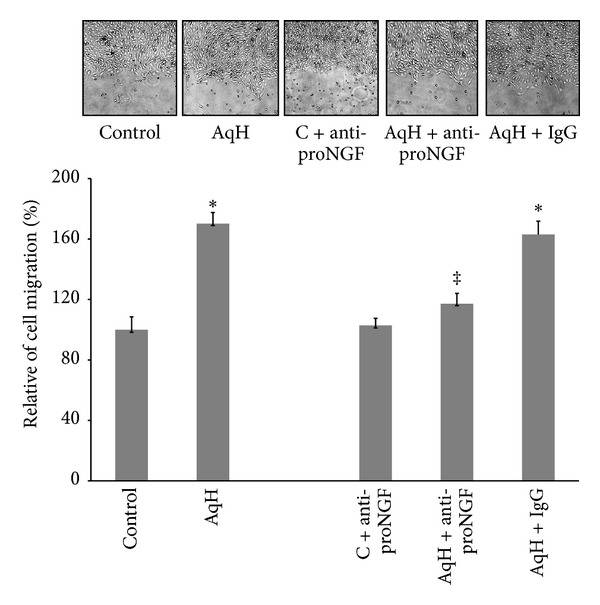
PDR-aqueous humor stimulates cell migration in a proNGF-dependent manner. HRE cells were grown to confluence and then scratched using a standard cell scrapper. Cells were switched to serum free medium and treated with aqueous humor samples (10 *μ*L/mL) in the presence or absence of either anti-proNGF antibody or rabbit IgG (1 *μ*g/mL). Representative micrographs for wounded HME cells are shown after 18 hours of various treatments. Statistical analysis showed that aqueous humor increased mean cell migration by 1.8-fold compared to the control group. Addition of anti-proNGF antibody to aqueous humor samples significantly reduced the mean percent of cell migration to the level of the control group whereas IgG did not significantly impact stimulatory effect of aqueous humor samples. Addition of anti-ProNGF antibody to control HRE cells did not significantly affect percent cell migration compared to control group (^∗,‡^statistically significant compared to control and aqueous humor groups, resp., (*P* < 0.05), *n* of aqueous humor samples = 7, *n* of cell cultures = 14–16).

Our previous studies have shown that diabetes-induced oxidative stress disturbs the homeostasis of nerve growth factor (NGF) resulting in accumulation of its precursor proNGF at the expense of the mature NGF in diabetic rat [11] and ocular fluids from diabetic patients [10]. Interestingly, the accumulation of proNGF was positively correlated with severity of diabetic retinopathy, where patients identified with proliferative diabetic retinopathy (PDR) showed higher levels (5-fold) of proNGF compared to nondiabetic samples [10]. Here, we examine the angiogenic response of aqueous humor samples from PDR patients using human retinal endothelial (HRE) cells in the presence or absence of anti-proNGF antibody (1 *μ*g/mL). Each aqueous humor sample (total of 100 *μ*L) was tested at least in duplicates on HRE cell culture (*n* = 7). As shown in Figure 1, treatment of HRE cells with PDR-aqueous humor significantly stimulated cell migration by 1.7-fold compared to the control group. Prior treatment of aqueous humor samples with anti-proNGF antibody significantly reduced the stimulatory effect of untreated-aqueous humor on cell migration to 1.2-fold of the control level. Whereas prior treatment with the isotope IgG maintained stimulatory effect (1.6-fold) of aqueous humor on cell migration. Treatment of control HRE cells with anti-proNGF antibody did not significantly impact cell migration compared to untreated control group. 

### 3.2. PDR-Aqueous Humor Stimulates Tube-Like Structures in a ProNGF-Dependent Manner

**Figure 2:**
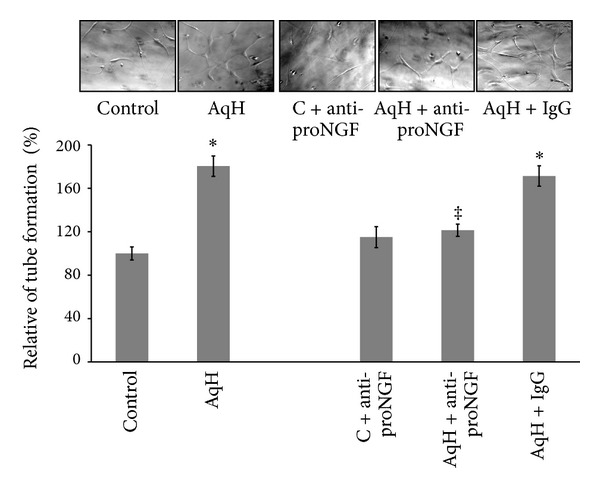
PDR-aqueous humor stimulates tube-like structures in a proNGF-dependent manner. HRE cells were grown into confluence then trypsinized and mixed with reduced-growth factor Matrigel and treated with aqueous humor samples (10 *μ*L/mL) in the presence or absence of either anti-proNGF antibody or rabbit IgG (1 *μ*g/mL). Representative micrographs for alignment of HRE into tube-like structures are shown after 18 hrs of incubation. Statistical analysis of tube length showed that aqueous humor increased mean tube formation 1.7-fold compared to the control group. Addition of anti-proNGF antibodies to aqueous humor samples significantly reduced the relative mean tube length but did not affect control group. Prior treatment of humor samples with rabbit IgG did not significantly reduce relative mean length when compared to the untreated aq. humor group (^∗,‡^statistically significant compared to control and aqueous humor groups, resp., (*P* < 0.05), *n* of aqueous humor samples = 7, *n* of cell cultures = 14–16).

We next examined the effects of PDR-aqueous humor on alignment of endothelial cells to tube-like structures. As shown in Figure 2, aqueous humor from PDR patients increased the relative mean tube length by 1.75-fold compared to the control group (*n* = 7). Prior treatment of aqueous humor samples with anti-proNGF antibody (1 *μ*g/mL) blunted the stimulatory effect of aqueous humor on inducing tube formation whereas IgG did not significantly affect tube formation. Meanwhile, treatment of control cells with anti-proNGF antibody did not markedly reduce tube formation compared to untreated control group. 

### 3.3. PDR-Aqueous Humor Stimulates Cell Proliferation in a ProNGF-Dependent Manner

**Figure 3:**
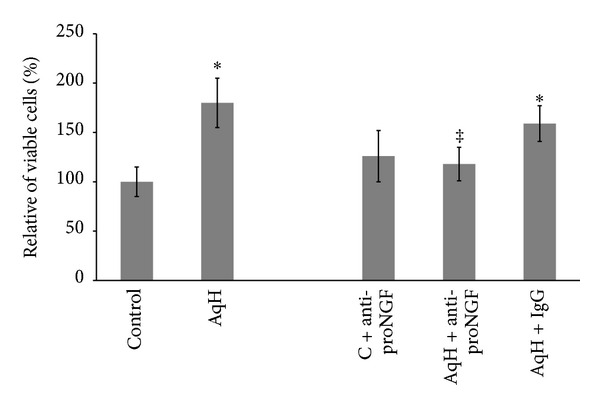
ProNGF and aqueous humor from PDR patients stimulated cell proliferation. HRE cells were grown into confluence then trypsinized and plated as described in method section. Cells were switched to serum free medium and treated with aqueous humor samples (10 *μ*L/mL) in the presence or absence of either anti-proNGF antibody or rabbit IgG (1 *μ*g/mL) for 24 hours then cells were trypsinized and counted. Statistical analysis showed that aqueous humor from PDR patients stimulated cell proliferation by 1.8-fold compared to the control group. Adding anti-proNGF antibody to aqueous humor samples significantly reduced the relative number of proliferating cells while IgG did not. Addition of anti-proNGF antibody to HRE cells did not affect number of proliferating cells (^∗,‡^statistically significant compared to control and aqueous humor groups, resp., (*P* < 0.05), *n* of aqueous humor samples = 6, *n* of cell cultures = 12–14).

We next examined the effect of aqueous humor on HRE cell proliferation. As shown in Figure 3, PDR-aqueous humor stimulated cell proliferation by 1.8 compared to the control group (*n* = 7). Prior treatment of aqueous humor samples with anti-proNGF antibody (1 *μ*g/mL) blunted the stimulatory effect of aqueous humor on cell proliferation, whereas prior treatment with IgG did not markedly reduce cell proliferation. Treatment of control cells with anti-proNGF antibody did not affect relative number of proliferating cells compared to untreated control.

### 3.4. ProNGF Activates TrkA/p38 MAPK in Retinal Endothelial Cells

**Figure 4:**
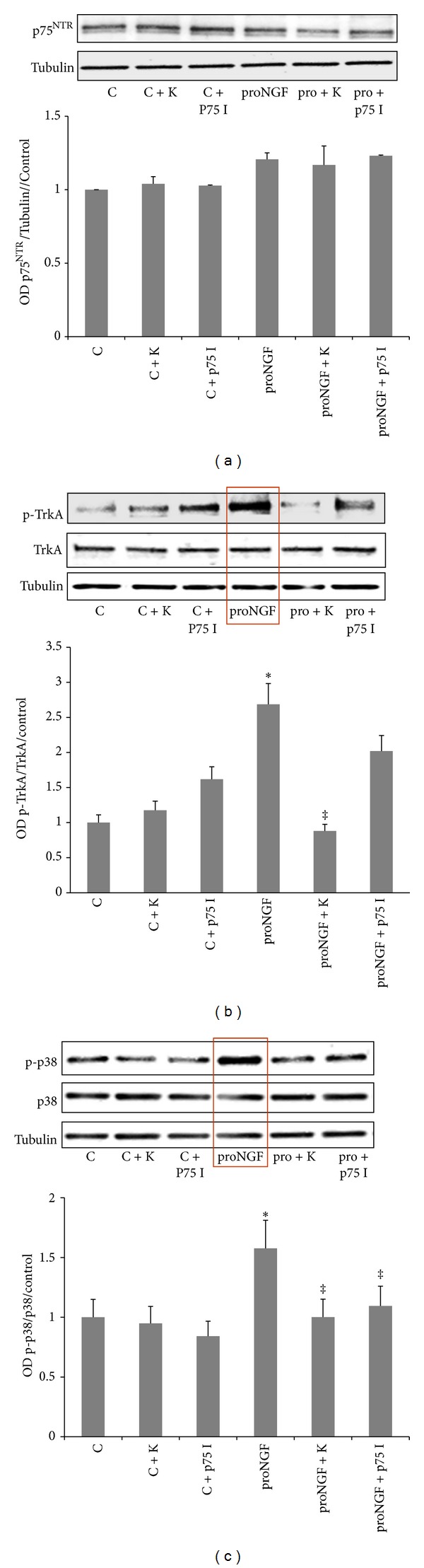
ProNGF activates TrkA/p38 MAPK in retinal endothelial cells. BRE cells were grown to subconfluence then switched to serum free medium and treated with mutant proNGF (50 ng/mL). Cells were harvested after 4 hours and subjected to western blot. (a) Representative image of p75^NTR^ and tubulin showing no significant change in p75^NTR^ expression among various groups. (b) Representative image and statistical analysis showed that proNGF is capable of activating TrkA in BRE cells compared to control cells. Treatment of BRE cells with the TrkA antagonist K252a abolished the ability of proNGF to activate TrkA while pharmacological inhibition of p75^NTR^ modestly increased TrkA activation. (c) Representative image and statistical analysis showed that proNGF activates p38 MAPK compared to controls. Inhibiting TrkA or p75^NTR^ abolished the ability of proNGF to activate p38 MAPK (^∗,‡^statistically significant compared to control and proNGF groups, resp., (*P* < 0.05), *n* = 3–5).

Previous studies showed that Trk receptors play a key role in mediating the mitogenic and angiogenic response of neurotrophins including NGF and BDNF [17, 25, 26]. Our previous work demonstrated significant upregulation of p75^NTR^ receptor expression in clinical and experimental diabetes [11, 27]. Therefore, we examined the impact of proNGF on activating TrkA and p75^NTR^ receptors in endothelial cells. As shown in Figure 4(a), there was no significant difference in p75^NTR^ expression among various groups. As shown in Figure 4(b), treatment of BRE cells with proNGF (50 ng/mL) stimulated phosphorylation of TrkA. Prior treatment of BRE cells with the TrkA antagonist K252a (0.1 *μ*M) blocked proNGF-mediated TrkA activation confirming the possibility that proNGF can activate TrkA. Interestingly, inhibition of p75^NTR^ using a selective antagonist modestly increased TrkA activation in both control and proNGF-stimulated cells suggesting mutual regulation of the two receptors TrkA and p75^NTR^. We next examined activation of p38 MAPK, and the results showed that proNGF activated p38 MAPK and this effect was abolished with TrkA antagonist (Figure 4(c)). Prior treatment of BRE cells with p75^NTR^ antagonist (20 *μ*M) significantly reduced proNGF-mediated activation of p38 MAPK (Figure 4(c)). These results suggest that proNGF can activate the mitogenic p38 MAPK signal in retinal endothelial cells.

### 3.5. Inhibiting TrkA Prevents ProNGF-Mediated Retinal Endothelial Cell Proliferation

**Figure 5:**
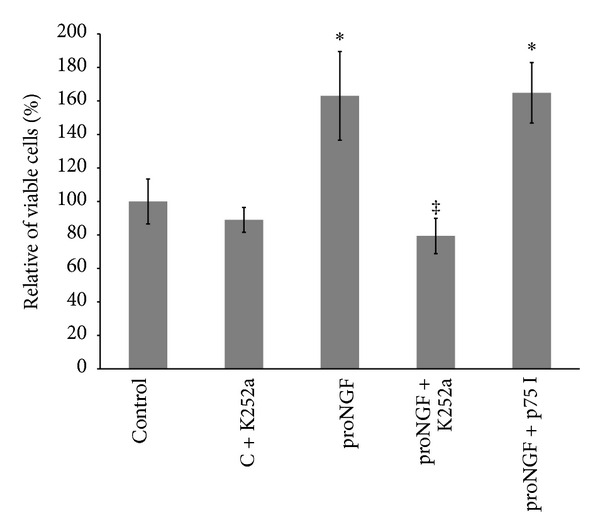
Inhibiting TrkA prevents proNGF-mediated retinal endothelial cell proliferation. BRE cells were grown into confluence and then trypsinized and plated as described in method section. Cells were switched to serum free medium and treated with mutant proNGF (50 ng/mL) in the presence or absence of K252a, TrkA inhibitor (0.1 *μ*M), or p75^NTR^ inhibitor (20 *μ*M) for 24 hours and then cells were trypsinized and counted. Statistical analysis showed that proNGF increased the percentage of proliferated cells by 1.6-fold compared to the control group. This effect was blocked by the specific TrkA receptor antagonist (K252a) but not with p75 inhibitor (^∗,‡^statistically significant compared to control and proNGF groups, resp. (*P* < 0.05), *n* = 5–7).

We next attempted to examine the effects of inhibiting TrkA on the mitogenic and angiogenic function of proNGF. As shown in Figure 5, treatment with the mutant proNGF (50 ng/mL) induced cell proliferation (1.6-fold) compared to untreated control. This effect was blocked by the specific TrkA receptor antagonist K252a (0.1 *μ*M), meanwhile, it was not reduced by p75^NTR^ inhibitor (20 *μ*M). Inhibition of TrkA in control group did not significantly inhibit cell proliferation compared to untreated controls.

### 3.6. Inhibiting TrkA Prevents ProNGF-Mediated Retinal Endothelial Cell Migration

**Figure 6:**
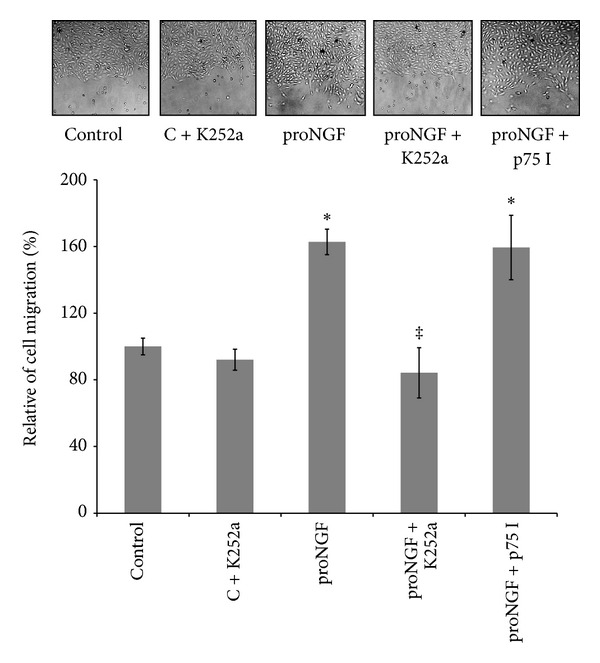
Inhibiting TrkA prevents proNGF-mediated retinal endothelial cell migration. BRE cells were grown to confluence and then scratched using a standard cell scrapper. Cells were switched to serum free medium and treated with mutant proNGF (50 ng/mL) in the presence or absence of either TrkA receptor antagonist, K252a (0.1 *μ*M), or p75 inhibitor (20 *μ*M). Representative micrographs for wounded BRE cells are shown after 18 hours of various treatments. Statistical analysis showed that proNGF increased mean cell migration by 1.98-fold compared to the control group. This effect was blocked by the specific TrkA receptor antagonist (K252a) but not with p75 inhibitor (^∗,‡^statistically significant compared to control and proNGF groups, resp., (*P* < 0.05), *n* = 6).

As shown in Figure 6, proNGF (50 ng/mL) increased the relative percentage of BRE cell migration by 1.8-fold compared to the control group. These effects were blocked with the specific TrkA receptor antagonist K252a (0.1 *μ*M), but not with p75^NTR^ inhibitor (20 *μ*M). Inhibition of TrkA in control group did not significantly inhibit cell migration compared to untreated controls.

### 3.7. Inhibiting TrkA Prevents ProNGF-Mediated Retinal Endothelial Tube Formation

**Figure 7:**
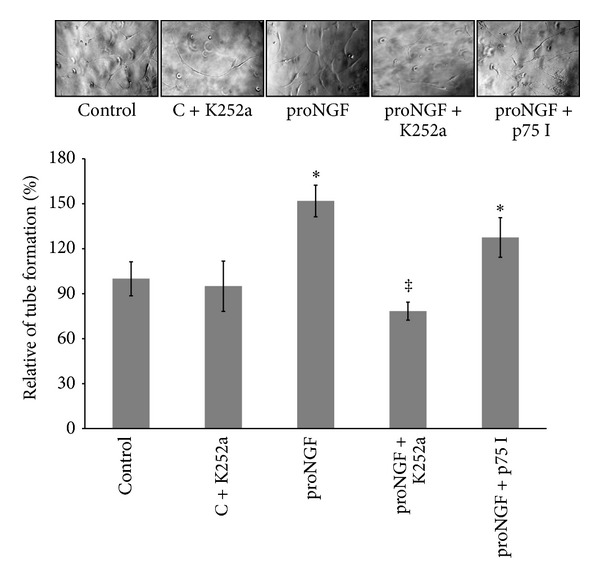
Inhibiting TrkA prevents proNGF-mediated retinal endothelial cell tube formation. BRE cells were grown into confluence and then trypsinized and mixed with reduced-growth factor Matrigel and treated with mutant proNGF (50 ng/mL) in the presence or absence of either TrkA receptor antagonist, K252a (0.1 *μ*M), or p75^NTR^ inhibitor (20 *μ*M). Representative micrographs for alignment of BRE into tube-like structures are shown after 18 hrs of incubation. Statistical analysis of tube length showed that proNGF increased mean tube formation 1.5-fold compared to the control group. This effect was blocked by the specific TrkA receptor antagonist (K252a) but not with p75 inhibitor (^∗,‡^statistically significant compared to control and proNGF groups, resp., (*P* < 0.05), *n* = 6).

As shown in Figure 7, proNGF (50 ng/mL) stimulated alignment of BRE cells into tube-like structures and tube length by 1.5-fold compared to the control group. This effect was blocked by the specific TrkA receptor antagonist K252a (0.1 *μ*M), but not with p75^NTR^ inhibitor (20 *μ*M). Inhibition of TrkA in control group did not significantly inhibit tube formation compared to untreated controls.

## 4. Discussion

Although increases in cytokines and growth factors including VEGF, TNF-*α*, IL-1*β*, and IL-6 have been well documented in vitreous from diabetic patients [28–30], little is known about the role of proNGF in PDR. The current study was conducted to evaluate the contribution of proNGF to the angiogenic response and to identify the possible underlying mechanisms. The main findings of the current study are that aqueous humor samples from PDR patients stimulate the angiogenic response in HRE cells in a proNGF-dependent manner and that exogenous proNGF mediates proangiogenic action via activation of TrkA/p38 MAPK pathway in retinal endothelial cells. We believe that this study is the first one to demonstrate evidence that proNGF can contribute to PDR and to provide insight into the possible mechanism. Future studies are warranted to further elucidate the complex role of proNGF in angiogenesis. 

Angiogenesis/neovascularization can be detrimental in pathological diseases, including diabetic retinopathy, arthritis, and tumor growth, as well as beneficial during wound healing and postischemic repair (reviewed in [31, 32]). Under diabetic conditions, prooxidative stress and pro-inflammatory milieu stimulate apoptosis of retinal vascular endothelial cells and capillary drop out leading to ischemia [6]. Normally to counteract the ischemic condition and salvage injured ischemic tissue, growth of collateral arteries from preexisting arterioles (reparative angiogenesis) is initiated [33]. This reparative mechanism appears to be impaired in the diabetic retina; however, in an effort to meet the metabolic demand of the retina, sprouting of capillaries and pathological neovascularization is triggered eventually leading to PDR. In response to ischemic stress, several growth factors including NGF are secreted to induce reparative angiogenesis via activation of TrkA receptor, promoting endothelial cell survival and angiogenesis [34]. Prior studies detected NGF at mRNA level or utilized ELISA assays, both of which cannot distinguish NGF from its precursor, and showed a positive correlation of NGF with progression of PDR in human [9, 35] or experimental retinal neovascularization models [25]. NGF is secreted as precursor form (proNGF) that gets cleaved to the mature NGF. Our previous analyses showed that diabetes-induced oxidative stress disturbs the homeostasis of NGF by hampering the cleavage of proNGF resulting in accumulation of proNGF and reducing NGF levels in experimental [11] and ocular fluids from PDR patients [10]. Therefore, it is conceivable that the previously reported increases in NGF are mixed proNGF/NGF rather than NGF alone. So far, researchers have focused on studying angiogenic response of NGF in retinal endothelial cells [18, 25, 36–38]; however, until now no studies have evaluated the possible angiogenic action of proNGF. Therefore, we tested the hypothesis that accumulated proNGF contributes to angiogenic response elicited by ocular fluids from PDR patients. Treatment of HRE cells with aqueous humor samples from diabetic patients stimulated endothelial cell migration, cell proliferation, and tube formation, all of which were inhibited by prior treatment with anti-proNGF antibody but not with rabbit IgG, confirming that proNGF directly contributes to angiogenesis. These results are consistent with the concept that diabetes deprives the retina from the neurotrophic support of NGF and favors accumulation of pro-inflammatory proNGF that can contribute to pathological neovascularization and PDR. 

Neurotrophins including NGF, BDNF, and neurotrophin-3 (NT-3) have been extensively studied for their actions on the nervous system. However, recent studies demonstrated the effects of neurotrophins as pleiotropic modulators of wound healing and angiogenesis [13–15, 39, 40]. The angiogenic response was either mediated through direct activation of the corresponding tropomyosin kinase receptor such as TrkA and TrkB in endothelial cells or indirectly via paracrine effects from the release of angiogenic factors from other cells [14, 34, 40]. Our results clearly show that proNGF can induce early activation (within 4 hours) of TrkA in retinal endothelial cells without significant effect on p75^NTR^ expression (Figure 4). Our results also show that inhibiting TrkA activation blocked proNGF-induced angiogenic response in retinal endothelial cells (Figures 5–7). Our results lend further support to a recent study showing that the angiogenic effect of proNGF in cancer cells is exerted mainly via TrkA rather than p75^NTR^ receptor [41]. The inhibitory effect of K252a, staurosporine-related compound [42], on angiogenic response have been demonstrated in several studies [14, 15, 18, 25, 36–38], nevertheless, we believe that our results are the first to demonstrate involvement of TrkA activation in response to proNGF in retinal endothelial cells. 

Activation of TrkA leads to its phosphorylation at Tyr^490^, which recruits the adaptor proteins GRB2-associated binding protein-1 and SH2-containing protein, activating MAPK/ERK kinase, and promotes neurite and endothelial growth [43]. Our results showing that proNGF activates TrkA/p38 MAPK and that inhibition of TrkA significantly inhibited proNGF-mediated cell proliferation, migration, and tube formation lend further support to other studies of the role of TrkA/p38 MAPK promoting cell growth, migration, and invasion of cancer cells [44, 45]. A study in smooth muscle cells showed also that activation of p38 MAPK and ERK was necessary for TrkA-mediated cell proliferation [45]. 

The p75^NTR^ receptor, a member of the tumor necrosis factor (TNF) receptor superfamily [46], has multiple and cell-specific functions dependent on availability of ligands and coreceptors (reviewed in [47–49]). In the retina, p75^NTR^ is expressed predominately by Müller cells; however, stress can induce expression of p75^NTR^ in other retina cell types including retinal ganglion cells [50] and endothelial cells [26, 51]. ProNGF has great affinity to bind and activate p75^NTR^ along with the sortilin receptor to mediate cell death [52]. We and others have shown that upregulation of proNGF induces p75^NTR^-mediated retinal neurodegeneration [10, 11, 53, 54]; and inflammation [54, 55] as well as endothelial cell death [26, 51, 56]. Interestingly, in the present study, results showed that inhibition of p75^NTR^ modestly activated TrkA (Figure 4(a)) and did not significantly alter proNGF-induced angiogenic response in retinal endothelial cells (Figures 5–7). These results lend further support to previous work demonstrating that inhibition of p75^NTR^ contributes to endothelial cell survival and inhibition of apoptosis rather than angiogenic function [26, 56]. Recent studies showed that p75^NTR^ played critical role in guiding migration of neuronal precursor cells and repair of vasculature in ischemic stroke model [57, 58]. Another study showed that p75^NTR^ is required for nitric oxide production in pulmonary endothelial cells [59]. As such, the proNGF/p75^NTR^ pathway is more likely involved in paracrine effects of other retina cell types in the diabetic retina rather than direct angiogenic process within endothelial cells. Further studies warrant characterization of the complex signaling pathway of proNGF/p75^NTR^ using in vivo models of retinal angiogenesis.

## Figures and Tables

**Table 1:** Clinical characteristics of participants providing aqueous humor samples.

	Sample 1	Sample 2	Sample 3	Sample 4	Sample 5	Sample 6	Sample 7
Gender	F	F	F	M	M	F	M
Race	Black	Black	White	White	Black	White	Black
Years of DM	20	26	17	18	16	28	19
